# Retinal small vessel narrowing in women with gestational diabetes, pregnancy-associated hypertension, or small-for-gestational age babies

**DOI:** 10.3389/fmed.2023.1265555

**Published:** 2023-10-16

**Authors:** Joseph Phillipos, Thao Vi Luong, Deborah Chang, Suresh Varadarajan, Paul Howat, Lauren Hodgson, Deb Colville, Judy Savige

**Affiliations:** ^1^The University of Melbourne (Northern Health and Melbourne Health), Parkville, VIC, Australia; ^2^Endocrine Unit, Northern Health, Epping, VIC, Australia; ^3^Department of Obstetrics and Gynaecology, Northern Health, Epping, VIC, Australia; ^4^University of Melbourne, Royal Victorian Eye and Ear Hospital East Melbourne, East Melbourne, VIC, Australia

**Keywords:** gestational diabetes, pregnancy-associated hypertension, small-for-gestational age babies, microvascular, placenta vessels, pregnancy, retinal vessel calibre

## Abstract

**Background:**

Gestational diabetes, pregnancy-associated hypertension and small-for-gestational age babies are all associated with impaired placental vascularisation. This study compared the effects of these conditions the systemic small vessel calibre that was examined in the retina.

**Methods:**

This was a cross-sectional observational study of consecutive pregnant women recruited from an antenatal clinic. Participants underwent a Glucose Tolerance Test, BP measurements, and were examined for small-for-gestational age babies as per national guidelines. They also underwent retinal photography with a non-mydriatic camera, and vessel calibres were measured with a validated semi-quantitative system at a retinal grading centre. Some participants also underwent testing of retinal vascular responsiveness to a flickering light.

**Results:**

Women with gestational diabetes (*n* = 68) had a higher mean arterial pressure (85 ± 9 mm Hg) than normal pregnant women (*n* = 27, 80 ± 8 mmHg, *p* = 0.01). They also had smaller mean retinal arteriole (147.5 ± 13.6 μm and 159.7 ± 6.7 μm respectively, *p* < 0.01) and venular calibre (221.0 ± 13.4 μm and 232.8 ± 20.1 μm respectively, *p* < 0.01) than normal. However their babies’ mean birth weights were not different from normal (3,311 ± 558 g and 3,401 ± 600 g respectively, *p* = 0.48). They also demonstrated a trend to reduced retinal arteriolar dilatation (3.5 ± 1.3%, *n* = 23) in response to vasodilatory stimuli (4.4 ± 1.8%) (*n* = 11) (*p* = 0.08) consistent with endothelial dysfunction. Women with pregnancy-associated hypertension (*n* = 35) had a higher mean arterial pressure (101 ± 12 mm Hg, *p* < 0.01), a smaller mean retinal arteriolar calibre (139.9 ± 10.6 μm, *p* < 0.0001), and a lower baby mean birth weight than for normal pregnancies (3,095 ± 443 g, *p* = 0.02). Likewise, women with small-for-gestational age babies (*n* = 31) had a higher mean arterial pressure (89 ± 19 mm Hg, *p* = 0.03), a smaller mean retinal arteriolar calibre (141.6 ± 12.8 μm, *p* < 0.01) and a lower baby mean birth weight than for normal pregnancies (2,468 ± 324 g, *p* < 0.0001).

**Conclusion:**

Mean retinal arterial calibre was reduced in women with gestational diabetes, pregnancy-associated hypertension or small-for-gestational age babies. The reduction in calibre was greatest in pregnancy-associated hypertension and small-for-gestational age babies. Systemic arteriole narrowing may contribute to the pathogenesis of placental vascular dysfunction in these conditions.

## Introduction

Normal pregnancy involves growth of foetal cytotrophoblasts into the maternal spiral arteries, replacing the endothelial cells and causing loss of the musculoelastic media (1, 2). This transforms the spiral arteries to high flow, low resistance vessels with decreased responsiveness to vasoactive substances, but with a normally increased blood supply to the developing foetus.

Both gestational diabetes and pregnancy-associated hypertension are characterised by abnormal placental vascularisation and metabolic adaptation, leading to reduced placental blood flow (3). This may be a mechanism in small-for-gestational age babies too.

The placental circulation comprises mainly small vessels. The retinal microvasculature represents a model for the systemic microvasculature *in vivo* including placental small vessels. This study directly compares retinal vessel calibre in women with the three major complications of pregnancy – gestational diabetes, pregnancy-associated hypertension, and small-for-gestational age babies, with the calibres in normal pregnancies.

Retinal imaging is non-invasive, convenient and reproducible. Both the conventional diabetic (4) and hypertensive microvascular grading systems (5), as well as small vessel calibre measurements (6) can be used to assess microvascular structural features. Gestational diabetes (7) and pregnancy-associated hypertension (8) have both been associated with retinal small vessel narrowing previously. Vessel calibre in the mothers of small-for-gestational age babies has not been examined before. Directly assessing small vessel changes in these diseases potentially increases our understanding of the relative effects of each condition. This study also looked at the overlap between these diagnoses, for example, how often pregnancy-associated hypertension occurred in gestational diabetes, and how often small-for-gestational age babies were found in each group. Overlap potentially complicates the interpretation of calibre changes.

In addition, the function of the retinal microvasculature may be studied using a dynamic vessel analyser, which is a retinal camera that records the response of the vessel calibre to flickering light. Normal vessels respond by transiently dilating, and returning to their baseline calibre when the flashes cease, probably due to the endothelial release of the local vasodilator, nitric oxide (9). Reduced dilatation reflects endothelial dysfunction, and has been studied previously in non-pregnant individuals with diabetes (10, 11) or with coronary artery disease (12). Here we have used the dynamic vessel analyser to examine responsiveness in gestational diabetes.

## Methods

### Overall study design

This was a single centre, cross-sectional, observational study of consecutive women recruited with gestational diabetes, pregnancy-associated hypertension, small-for-gestational age babies, or normal pregnancies. The aim of this study was to determine and compare the changes in retinal small vessel calibre in women with gestational diabetes in trimester 3, pregnancy-associated hypertension, small-for-gestational age babies or normal pregnancies.

Participants provided medical details, then underwent BP measurement and retinal imaging, and their records were reviewed after delivery to determine their babies’ birth weights and any further complications. Participants’ deidentified retinal photographs were then examined for arteriole and venular calibre at a retinal grading centre, and the calibre measurements compared between women with gestational diabetes, pregnancy-associated hypertension, small-for-gestational age babies and those with normal pregnancies. In addition, the small vessel responses to a flashing light were assessed using a dynamic vessel analyser, in women with gestational diabetes or normal pregnancies.

The study was approved by the Northern Health Human Research Ethics Committee according to the Principles of Helsinki, and all participants provided signed, informed consent.

### Participants

Consecutive women with gestational diabetes, pregnancy-associated hypertension, small-for-gestational age babies, or normal pregnancies were recruited from the medical obstetrics or antenatal clinics of a metropolitan teaching hospital over two 6-month periods. All attendees with gestational diabetes in trimester 3 were approached to participate. Exclusion criteria were age under 18 years, known type I or type II diabetes, twin pregnancies, or ungradable retinal images. Some women were also studied in trimester 2 or within 3 days postpartum.

Pregnant women routinely undergo a glucose tolerance test at 24 to 28 weeks’ gestation where gestational age is assessed by an obstetrician using standard ultrasound criteria. Gestational diabetes was identified with an abnormal test as described by the Australian Diabetes in Pregnancy Society (ADIPS) (13), with a glucose level fasting >5.1 mmol/L, >10.0 mmol/L after 1 hour or >8.5 mmol/L after 2 hours. Pregnancy-associated hypertension was diagnosed when the BP was >140/90 mm Hg according to the Society of Obstetric Medicine of Australian and New Zealand (SOMANZ) guidelines.^1^ Small-for gestational- age babies were defined by a foetal weight at delivery below the 10th percentile for gestational age. However these were often predicted during pregnancy at which time the mothers were invited to undergo retinal imaging.

Controls were normal pregnant women who did not have twins, gestational diabetes, pregnancy-associated hypertension or small for gestational age babies. Overlapping diagnoses, for example, gestational diabetes and pregnancy-associated hypertension or small- for-gestational dates, were noted in order to ascertain the effect of a single diagnosis on retinal small vessel calibre.

Participants were assisted to complete a structured questionnaire that included age, smoking and hypertension. They were rested and a clinic BP measured using a Hg sphygmomanometer. Recruitment, data capture, BP measurements and retinal imaging were coordinated at a single clinic visit. Charts were revisited after delivery to check for further medical details.

### Measurements

#### Retinal imaging and calibre measurements

Participants underwent retinal colour photography of both eyes using a non-mydriatic retinal camera (CANON, Japan). Standard 45° images were taken, with at least one centred on the macula and another on the optic disc.

Retinal vessel calibre was measured by a trained grader at a retinal grading centre (Royal Victoria Eye and Ear Hospital, Melbourne, Australia) using a standardized protocol for grading the digital retinal images (14, 15). All vessels passing through a zone 0.5–1 disc diameters from the optic disc margin were examined using a semi-automated computer imaging program (University of Wisconsin, WI), and measures based on the 6 largest vessels were combined into the Central Retinal Artery and Vein Equivalents (CRAE and CRVE) (16), using a computer-assisted method and Knudtson’s modification of the Parr-Hubbard formula (14, 15). This technique was highly reproducible with high intra- and inter-class correlation coefficients (17).

#### Dynamic vessel analyser

The subject’s non-dominant eye was dilated with 1% tropicamide (Bausch and Lomb, United States). The subject focused on a fixed point and the camera (Dynamic Vessel Analyser, IMEDOS, Germany) was centred on the subject’s retina according to a previous protocol (11, 18). Arterioles and venules in the superior temporal region were selected to measure calibre. The subject underwent a total of 350 s of testing, comprising three sets of flickering light each of 20 s. Testing began at 50, 150, and 250 s. During the non-flicker period, a constant green light allowed the machine to measure the baseline calibre. At the conclusion of testing, the software generated the mean percentage increase in vessel calibre in response to the flickering light from the three experiments.

#### Statistical analysis

Retinal microvascular calibres were compared using Fisher’s exact test or the student *t*-test. Odds ratios were used to characterise dichotomous data and an association was considered significant if the odds ratio was >1.00, and the 95% confidence interval did not include 1.00. The data were also examined using linear regression. Statistical analyses were performed using STATA v.11.2 software (Stata Corp, College Station, TX, United States). A *p* value <0.05 was considered significant and a *p* value <0.10 was considered a trend.

## Results

### Patient recruitment

This was a cross-sectional study where each patient was usually examined once only. Normal pregnant women were recruited in trimester 2 (*n* = 39), trimester 3 (*n* = 27), or post-partum (*n* = 28).

Women with gestational diabetes were recruited after routine screening in pregnancy in trimester 2 (*n* = 18), trimester 3 (*n* = 68), or post-partum (*n* = 39).

Women with pregnancy-associated hypertension were recruited in trimester 3 (*n* = 35) because pregnancy-associated hypertension usually has its onset in the third trimester. Women with small-for-gestational age babies were also recruited in trimester 3 (*n* = 31) because gestational diabetes is usually detected in the third trimester.

### Gestational diabetes

Sixty-eight women with gestational diabetes in trimester 3 were compared with 27 normal pregnant women also in trimester 3 (Table 1). The women with gestational diabetes had a mean age of 31.1 ± 4.8 years (*p* = 0.13), and a mean weight of 85.7 ± 22.2 kg (*p* = 0.16). Five had previously diagnosed hypertension (7.4%, *p* = 0.3), and four developed pregnancy-associated hypertension (5.9%, *p* = 0.37). They demonstrated trends to a shorter mean gestation period (*p* = 0.07) and more small-for-gestational age babies (*n* = 7, *p* = 0.09). However overall the women with gestational diabetes had babies with a mean birth weight that was not different from that for normal pregnancies (3,311 ± 558 g and 3,401 ± 600 g respectively, *p* = 0.48).

**Table 1:** Clinical features in women with gestational diabetes, pregnancy-associated hypertension, small-for-gestational age babies or normal pregnancies in the third trimester.

Clinical feature	Non-diabetic pregnant controls (*n* = 27)	Gestational diabetes (*n* = 68)	*p* value, OR, 95%CI compared with controls	Pregnancy-associated hypertension (*n* = 35)	*p* value, OR, 95%CI compared with controls	Mothers of small-for-gestational age babies (*n* = 31)	*p* value, OR, 95%CI compared with controls
Age (years)	29.5 ± 4.9	31.1 ± 4.8	*p* = 0.13	29.8 ± 6.0	*p* = 0.77	28.9 ± 5.5	*p* = 0.65
Weight (Kg)	75.9 ± 15.2	85.7 ± 22.2	*p* = 0.16	93.7 ± 23.6	***p* < 0.01**	78.1 ± 21.8	*p* = 0.66
Mean arterial pressure (mean, SD, mmHg)	80 ± 8	85 ± 9	*p* = **0.01**	**101** ± **12**	***p* < 0.01**	**89** ± **19**	***p* = 0.03**
Systolic BP (mean, SD, mmHg)	106 ± 13	115 ± 11	***p* < 0.01**	133 ± 15	***p* < 0.0001**	**116** ± **23**	***p* = 0.05**
Diastolic BP (mean, SD, mmHg)	67 ± 7	71 ± 10	*p* = 0.98	86 ± 11	***p* < 0.0001**	75 ± 18	***p* = 0.03**
Previous hypertension	0	5 (7.4%)	*p* = 0.3, OR = 4.8, 0.25 to 89	4 (11.4%)	*p* = 0.17, OR = 7.9, 0.4 to 152	1 (3.2%)	*p* = 0.55, OR = 2.7, 0.1 to 69.1
Smoking history	7 (37%)	19 (28%)	*p* = 0.84, OR = 1.10, 0.4 to 3.0	11 (31%)	*p* = 0.64, OR 1.3, 0.4 to 4.0	7 (23%)	p = 0.77, OR = 0.83, 0.25 to 2.78
Mean number of previous pregnancies	2 ± 1.9	1.8 ± 1.7	*p* = 0.60	1.5 ± 1.6	*p* = 0.25	1.2 ± 1.4	***p* = 0.05**
Gestational diabetes	0	68 (100%)	*p* = 0.005, OR = 55, 3.3 to 919.9	7 (20%)	***p* = 0.07**, OR = 14.5, 0.79 to 266	8 (23%)	***p* = 0.04, OR 19.9, 1.1 to 363**
Pregnancy-associated hypertension	0	4 (5.9%)	*p* = 0.37, OR 3.8, 0.19 to 73.7	35 (100%)	*p* = 0006, OR = 55, 3.2 to 937	6 (19%)	*p* = 0.07, OR 0.75 to 261
Estimated gestation at delivery (mean, SD, weeks)	39.3 ± 1.5	38.7 ± 1.5	***p* = 0.07**	38.5 ± 1.7	***p* = 0.058**	**38.0** ± **1.6**	***p* = 0.002**
Birth weight (mean, SD, grams)	3,401 ± 600	3,311 ± 558	*p* = 0.48	3,095 ± 443	***p* = 0.02**	2,468 ± 324	***p* < 0.0001**
Small for gestational age baby	0	7 (10%)	*p* = 0.2, OR = 6.7, 0.4 to 121.6	6 (17%)	***p* = 0.09**, OR = 12.1, 0.65 to 225	31 (100%)	

Significant values are in bold type.

Women with gestational diabetes in trimester 3 had a higher mean arterial blood pressure (85 ± 9 mm Hg) than the normal pregnant women (80 ± 8 mm Hg, *p* = 0.01). They also had a smaller mean retinal arteriolar calibre than normal pregnant women (147.5 ± 13.6 μm and 159.7 ± 6.7 μm respectively, *p* < 0.01) and a smaller mean venular calibre (221.0 ± 13.4 μm and 232.8 ± 20.1 μm respectively, *p* < 0.01) (Table 2).

**Table 2:** Arteriolar and venular calibre in women with gestational diabetes, pregnancy-associated hypertension, small-for-gestational age babies or normal pregnancies.

Trimester	Arteriolar calibre			Venular calibre		
	Disease (μm)	Normal pregnancy (μm)	*p* value	Disease (μm)	Normal pregnancy (μm)	*p* value
Gestational diabetes						
Trimester 2	151.7 ± 13.7 (*n* = 18)	160.4 ± 7.4 (*n* = 39)	**<0.01**	228.8 ± 15.7 (*n* = 18)	234.6 ± 13.9 (*n* = 39)	0.16
Trimester 3	147.5 ± 13.6 (*n* = 68)	159.7 ± 6.7 (*n* = 27)	**<0.01**	221.0 ± 13.4 (*n* = 68)	232.8 ± 20.1 (*n* = 27)	**<0.01**
Postpartum	141.9 ± 13.6 (*n* = 39)	152.1 ± 15.1 (*n* = 28)	**<0.01**	217.6 ± 14.7 (*n* = 39)	229.8 ± 23.9 (*n* = 28)	**0.01**
Pregnancy-associated hypertension						
Trimester 3	139.9 ± 10.6 (*n* = 35)	159.9 ± 6.7 (*n* = 27)	**<0.0001**	211.7 ± 6.4 (*n* = 35)	232.8 ± 20.1 (*n* = 27)	**<0.0001**
Small-for-gestational age babies						
Trimester 3	141.6 ± 12.8 (*n* = 31)	159.7 ± 6.7 (*n* = 27)	**<0.01**	218.5 + 20.1 (*n* = 31)	232.8 ± 20.1 (*n* = 27)	**0.01**

Significant values are in bold type.

The diagnosis of gestational diabetes was associated with smaller arteriole calibre (−12.3 μm, 95% CI −17.75 to −6.86 um, *p* < 0.01) independent of age, mean arterial pressure, hypertension, and smoking history in a linear regression model (Table 3). Gestational diabetes was also associated with smaller venular calibre (−11.7 μm, 95% confidence level −18.82 to −4.55 um, *p* < 0.01) independent of age, mean arterial pressure, hypertension, and smoking history.

**Table 3:** Determinants of arteriolar and venular calibre in women with gestational diabetes in Trimester 3.

Variable	Co-efficient	95% confidence interval		*p* value
Arteriole calibre				
Age	−0.14	−0.68	0.41	0.89
Mean arterial pressure	−0.28	−0.55	0.00	0.28
Hypertension	−3.00	−15.04	9.02	0.9
Smoking	−2.08	−0.81	3.94	0.52
Gestational diabetes	−12.3	−17.75	−6.86	**<0.01**
Venular calibre				
Age	−0.58	−1.21	0.06	0.07
Mean arterial pressure	0	−0.33	0.33	1
Hypertension	−3.47	−0.33	0.33	0.62
Smoking	4.47	−2.51	11.45	0.21
Gestational diabetes	−11.7	−18.82	−4.55	**<0.01**

Significant values are in bold type.

Data from women with gestational diabetes in trimester 2 demonstrated that the reduction in arteriole and venular calibre found in trimester 3 had begun in trimester 2 (Table 2). The mean arteriolar calibre was 151.7 ± 13.7 μm (*n* = 18) in trimester 2 and 160.4 ± 7.4 μm (*n* = 39) in normal pregnant women at the same stage (*p* < 0.01). The corresponding mean retinal venular calibre was 228.8 ± 15.7 μm in gestational diabetes in trimester 2 and 234.6 ± 13.9 μm in normal pregnant women (*n* = 39, *p* = 0.16).

The reduction in arteriole calibre in gestational diabetes persisted post-partum. The mean arteriolar calibre after delivery was 141.9 ± 13.6 μm (*n* = 39) in women with gestational diabetes and 152.1 ± 15.1 μm in normal women post-partum (*n* = 28, *p* < 0.01). The corresponding mean retinal venular calibre was 217.7 ± 14.7 μm (*n* = 39) and 229.8 ± 23.9 μm in normal pregnant women (*n* = 28, *p* = 0.01).

### Endothelial dysfunction

**Figure 1:**
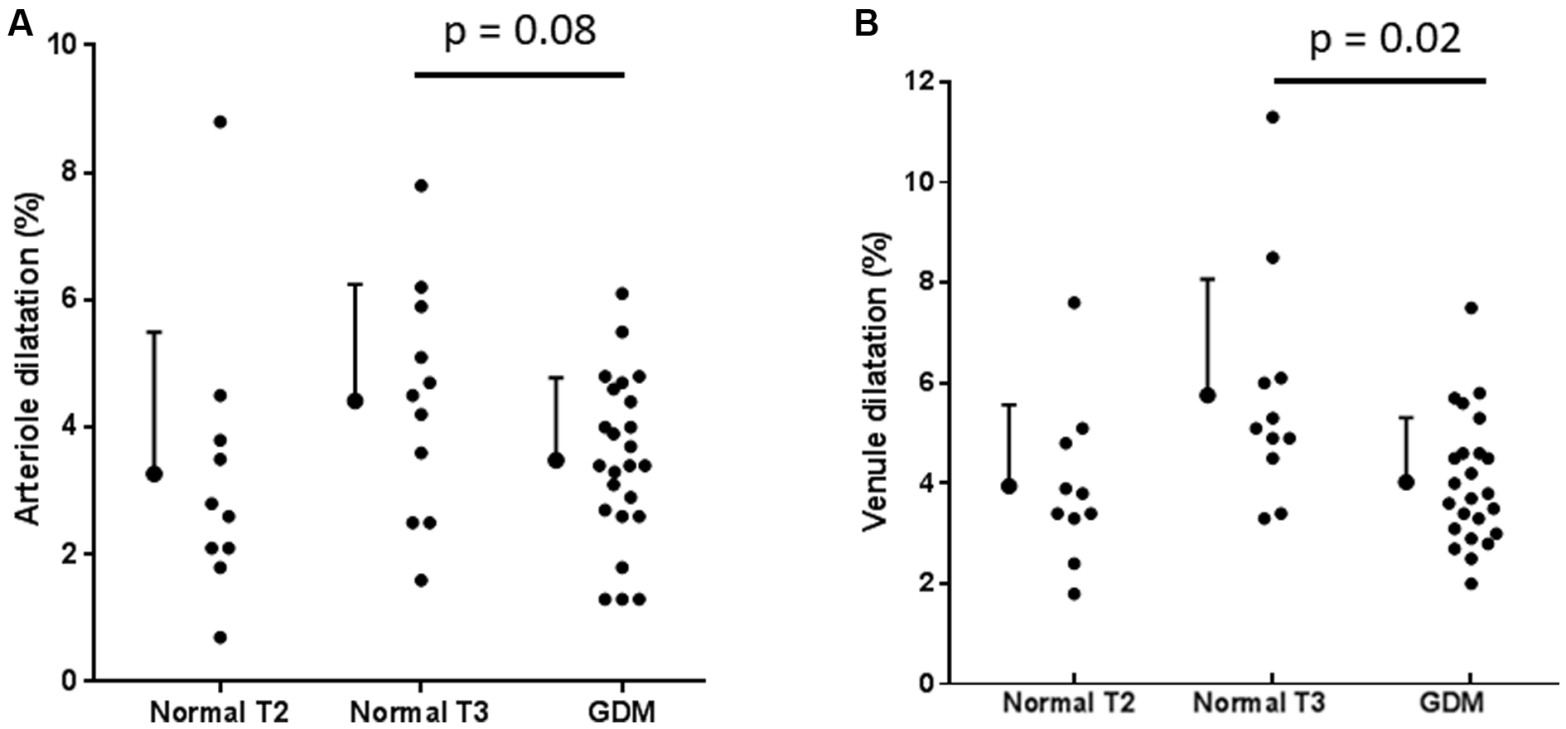
Arteriole **(A)** and venular **(B)** calibre in gestational diabetes (GDM) and normals in trimesters 2 (T2) and 3 (T3).

Dynamic vessel analysis in 24 of the women studied with gestational diabetes in trimester 3 demonstrated a trend to reduced arteriolar dilatation (3.5 ± 1.3%) compared with dilatation in 11 normal pregnant women in trimester 3 (4.4 ± 1.8%, *p* = 0.08) (Figure 1A). Venular dilatation was also less (4.0 ± 1.3%) than in normal women in trimester 3 (5.8 ± 2.3%, *p* = 0.02) consistent with endothelial dysfunction (Figure 1B).

### Pregnancy-associated hypertension

Thirty-five women with pregnancy-associated hypertension in trimester 3 were compared with 27 normal pregnant women in trimester 3 (Table 2). The women with pregnancy-associated hypertension had a mean age of 29.8 ± 6.0 years (*p* = 0.77) and weight of 93.7 ± 23.6 Kg (*p* < 0.01). They included four women with previously-diagnosed hypertension (11.4%), and 7 (20%) with gestational diabetes (*p* = 0.07). They demonstrated a trend to a shorter mean gestation period (*p* = 0.058), and more small-for-gestational age babies (*n* = 6, *p* = 0.09). Their babies’ mean birth weight was 3,095 ± 443 g which was less than in normal pregnancies (3,401 ± 600 g, *p* = 0.02).

The women with pregnancy-associated hypertension had a higher mean arterial pressure (101 ± 12 mm Hg) than normal pregnant women in trimester 3 (80 ± 8 mm Hg, *p* < 0.01) (Table 2). They also had a smaller mean arteriolar calibre than normal pregnancies (139.9 ± 10.6 μm, 159.9 + 6.7 μm respectively, *p* < 0.0001), and a smaller mean venular calibre (211.7 ± 6.4 μm, 232.8 ± 20.1 μm respectively, *p* < 0.0001).

The increase in mean arterial pressure in pregnancy-associated hypertension was greater than in gestational diabetes (diff 16, 12 to 12 mm Hg, *p* < 0.0001), the reduction in arteriole calibre was also greater (diff −7.6, −2.7 to −12 μm, *p* = 0.005), and their babies’ mean birth weight was less (*p* = 0.0494).

### Mothers of small-for-gestational age babies

Thirty-one women with small-for-gestational age babies were compared with the 27 normal pregnant women in trimester 3 (Table 1). The mean age of the women with small-for-gestational age babies was 28.9 ± 5.5 years (*p* = 0.65) and their mean weight was 78.1 ± 21.8 kg (*p* = 0.66). They included one woman with previously-diagnosed hypertension (3%), 8 with gestational diabetes (23%) and 6 with pregnancy-associated hypertension (19%). The women with small-for-gestational age babies had a shorter mean gestation period (*p* = 0.002), and their babies’ mean birth weight was 2,468 ± 324 g which was less than in normal pregnancies (3,401 ± 600 g, *p* < 0.0001).

The women with small-for-gestational age babies (*n* = 31) had a higher mean arterial pressure (89 ± 19 mm Hg) than normal pregnant women in trimester 3 (80 ± 8 mm Hg, *p* = 0.03) (Table 1). They also had a smaller mean retinal arteriolar calibre than normal pregnancies (141.6 ± 12.8 μm, 159.9 ± 6.7 μm respectively, *p* < 0.01) and a smaller mean venular calibre (218.5 ± 20.1, 232.8 ± 20.1 μm respectively, *p* < 0.01) (Table 2).

Women with small-for-gestational age babies had a mean arterial pressure increase of 4 mm Hg more than in gestational diabetes (diff 4.0, 1.6 to 9.6, *p* = 0.16), their reduction in arteriole calibre was greater (diff −5.9, −0.1 to −11.7, *p* = 0.046), and their babies’ mean birth weight was less than for gestational diabetes (2,468 ± 324 g, *p* < 0.0001).

### Comparison of retinal arterial calibre in different complications of pregnancy

Mean retinal arterial calibre was reduced in gestational diabetes, pregnancy-associated hypertension and mothers of small-for-gestational age babies in trimester 3, and the reduction was greatest in women with pregnancy-associated hypertension (*p* = 0.005) or small-for-gestational age babies (*p* = 0.04) compared with gestational diabetes (Table 2). There was no difference in arteriole calibre between pregnancy-associated hypertension and small-for-gestational age babies (*p* = 0.56). In addition, babies were smallest with pregnancy-associated hypertension and small-for-gestational age babies (Table 2).

## Discussion

In this study, women with gestational diabetes, pregnancy-associated hypertension or small-for-gestational age babies all had an increased mean arterial pressure and reduced retinal arteriole calibre compared with women with normal pregnancies. Hypertension in general is associated with reduced retinal small vessel calibre (19, 20). The arteriole narrowing in gestational diabetes was less pronounced than in pregnancy-associated hypertension and in women with small-for-gestational age babies, and their babies’ birth weight was not affected. Thus the increased mean arterial pressure was associated with a smaller arteriole calibre in all three conditions and associated with smaller babies in the two conditions with the higher mean arterial pressures. These observations suggest a relationship between the mean arterial pressure, arteriole narrowing, and birth weight.

Previous studies have correlated retinal arteriole calibre with the calibre in the systemic microvasculature including small vessels in the placenta which suggests that the reduced retinal arteriole calibre correlates directly with decreased foetal growth (21). The inability to demonstrate a direct relationship in this study between blood pressure and birthweight may have been because of confounders such as placental infarction or maternal smoking in the women with low birthweight babies. The lack of a relationship between the reduction in arteriole calibre and increased blood pressure in the linear regression model of gestational diabetes may have been because only one blood pressure measurement was recorded.

This study found that systemic arterioles and venules were narrowed in women with gestational diabetes in trimester 3. The narrowing was present from at least trimester 2, when the diagnosis of gestational diabetes was first made, and persisted post-partum. Other studies have also found that vascular calibre is reduced at an earlier stage than the diagnosis of gestational diabetes is suspected clinically (7). In addition, reduced vessel calibre was found as early as 13 weeks’ gestation in women who subsequently developed preeclampsia (22). Interestingly, in normal pregnancies, arteriolar calibre also decreased from trimester 2 to immediately post-partum but the reduction in calibre was less than in gestational diabetes, pregnancy-associated hypertension and women with small-for-gestational age babies in our study.

Our results confirmed previous reports that women with gestational diabetes have retinal arteriolar abnormalities including smaller calibre and a decreased vasodilator response to flickering light (23). A reduced arteriole calibre has also been observed in pregnant women with type I diabetes (24). The vessel pattern in gestational diabetes may be different too with reduced fractal dimensions and larger branching angles (7). While arteriolar narrowing may be an independent predictor of gestational diabetes (7) our study also demonstrated narrowing in pregnancy-associated hypertension and in mothers of small-for-gestational age babies. Already there are simple accepted reproducible criteria for the diagnosis of gestational diabetes, pregnancy-associated hypertension and small-for-gestational age babies and the demonstration of a smaller retinal arteriole calibre probably represents a model for a shared pathogenetic mechanism rather than a novel diagnostic tool.

The arteriolar narrowing in gestational diabetes is unusual because microvascular calibre is usually increased in diabetes not associated with pregnancy (25, 26), and venular, and to a lesser extent arteriolar, widening are common with any form of systemic inflammation including diabetes. In gestational diabetes the vasodilatory stimulus may have been countered by a stronger vasoconstrictive effect of the associated hypertension, masking any vasodilatation. Importantly this study demonstrated that the narrowing was not as pronounced as in the cohort of women with pregnancy-associated hypertension nor the women with small-for-gestational age babies.

Other methods used to investigate the microvasculature in pregnancy have included *ex vivo* analysis of maternal vessels (27) and ophthalmic artery Doppler ultrasonography (28, 29). Such studies have suggested deficient vasodilation in subjects with pregnancy-associated hypertension which was confirmed with our dynamic vessel analyser in gestational diabetes.

Gestational diabetes is associated with an insufficient placental blood flow. Increased placental vascular resistance is a homeostatic response to the diabetic environment, that reduces glucose supply to the foetus (30). In gestational diabetes the placental villous vessels have a smaller calibre, with enlarged endothelial cells further reducing the lumen diameter (31). A previous study found reduced small retinal vessel density in gestational diabetes using fractal dimensions (7). Decreased small vessel density in the placental bed also occurs in pregnancy-associated hypertension (32). Both the retina and placenta lack an autonomic innervation and blood flow depends on vessel calibre. Vascular tone is largely determined by endocrine and paracrine factors. Dilatation depends on endogenously produce nitric oxide and the reduced response to a flickering light in our functional assay reflects reduced endothelium-dependent vasodilation (33).

Although this study found that retinal microvascular changes were present even before the diagnosis of gestational diabetes there is no evidence that these changes were also present pre-pregnancy. Other studies of ours indicate that the retinal microvascular calibre of women with gestational diabetes was normal in the first trimester. Similarly, patients with preeclampsia had a normal blood pressure in their first trimester. However, interestingly, individuals with gestational diabetes have an increased risk of diabetes post-partum and their retinal vessels are more likely to be dilated at a 5 year review (23). There are also data that women with preeclampsia have an increased risk of reduced retinal small vessel calibre 5 years after delivery (34).

The main strengths of this study were the accuracy of the clinical data which took into account simultaneous pregnancy complications, and the reproducibility of the retinal vascular calibre measurements. This study compared, for the first time, vessel calibres in gestational diabetes, pregnancy-associated hypertension and mothers with small-for-gestational age babies, and ascertained the number of participants with dual diagnoses. Participants with dual diagnoses were however probably too few to affect the mean arterial pressure, arteriole calibre or birth weights of the overall cohort. Other studies of retinal vascular calibre should also record where there are two pregnancy complications simultaneously.

The study’s major limitations were its cross-sectional, observational nature, as well as the lack of a correlation between mean arterial pressure and arteriolar narrowing, possibly because only one blood pressure measurement was recorded. The number of study participants was consistent with previous reports (7, 21, 22, 35, 36).

In conclusion, women with gestational diabetes, pregnancy-associated hypertension, or small-for-gestational age babies all have an increased mean arterial pressure and reduced retinal arteriole calibre. Since retinal small vessel calibre represents a surrogate for systemic small vessel calibre, these findings suggest a shared pathogenesis and that retinal arteriole narrowing should be examined further as a model for impaired placental blood flow and smaller foetal size in these three conditions.

## Data availability statement

The raw data supporting the conclusions of this article will be made available by the authors, without undue reservation.

## Ethics statement

The studies involving humans were approved by Austin Health (by delegation from Northern Health) HREC/16/Austin/539. The studies were conducted in accordance with the local legislation and institutional requirements. The participants provided their written informed consent for involvement in the study. Written informed consent was obtained from the individual(s) for the publication of any potentially identifiable images or data included in this article.

## Author contributions

JP: Writing – review & editing, Data curation, Investigation. TL: Data curation, Investigation, Writing – review & editing. DCh: Data curation, Investigation, Writing – review & editing. SV: Data curation, Investigation, Writing – review & editing. PH: Investigation, Resources, Writing – review & editing. LH: Methodology, Resources, Software, Writing – review & editing. DCo: Methodology, Supervision, Writing – review & editing. JS: Conceptualization, Supervision, Writing – original draft, Writing – review & editing.

## Footnotes

^1^ https://www.somanz.org/content/uploads/2020/07/HTguidelineupdatedJune2015.pdf
